# Combining self-reported and objectively measured survey data to improve hypertension prevalence estimates: Portuguese experience

**DOI:** 10.1186/s13690-021-00562-y

**Published:** 2021-04-08

**Authors:** Irina Kislaya, Andreia Leite, Julian Perelman, Ausenda Machado, Ana Rita Torres, Hanna Tolonen, Baltazar Nunes

**Affiliations:** 1 Departament of Epidemiology, National Health Institute Doutor Ricardo Jorge, Lisbon, Portugal; 2 NOVA National School of Public Health, Public Health Research Centre, Universidade NOVA de Lisboa, Lisbon, Portugal; 3 Comprehensive Health Research Center (CHRC), Universidade NOVA de Lisboa, Lisbon, Portugal; 4 Department of Public Health and Welfare, Finnish Institute for Health and Welfare (THL), Helsinki, Finland

**Keywords:** Hypertension, Self-reports, Misclassification error, Bias correction, Multiple imputation, Survey, MIME

## Abstract

**Background:**

Accurate data on hypertension is essential to inform decision-making. Hypertension prevalence may be underestimated by population-based surveys due to misclassification of health status by participants. Therefore, adjustment for misclassification bias is required when relying on self-reports. This study aims to quantify misclassification bias in self-reported hypertension prevalence and prevalence ratios in the Portuguese component of the European Health Interview Survey (INS2014), and illustrate application of multiple imputation (MIME) for bias correction using measured high blood pressure data from the first Portuguese health examination survey (INSEF).

**Methods:**

We assumed that objectively measured hypertension status was missing for INS2014 participants (*n* = 13,937) and imputed it using INSEF (*n* = 4910) as auxiliary data. Self-reported, objectively measured and MIME-corrected hypertension prevalence and prevalence ratios (PR) by sex, age group and education were estimated. Bias in self-reported and MIME-corrected estimates were computed using objectively measured INSEF data as a gold-standard.

**Results:**

Self-reported INS2014 data underestimated hypertension prevalence in all population subgroups, with misclassification bias ranging from 5.2 to 18.6 percentage points (pp). After MIME-correction, prevalence estimates increased and became closer to objectively measured ones, with bias reduction to 0 pp - 5.7 pp. Compared to objectively measured INSEF, self-reported INS2014 data considerably underestimated prevalence ratio by sex (PR = 0.8, 95CI = [0.7, 0.9] vs. PR = 1.2, 95CI = [1.1, 1.4]). MIME successfully corrected direction of association with sex in bivariate (PR = 1.1, 95CI = [1.0, 1.3]) and multivariate analyses (PR = 1.2, 95CI = [1.0, 1.3]). Misclassification bias in hypertension prevalence ratios by education and age group were less pronounced and did not require correction in multivariate analyses.

**Conclusions:**

Our results highlight the importance of misclassification bias analysis in self-reported hypertension. Multiple imputation is a feasible approach to adjust for misclassification bias in prevalence estimates and exposure-outcomes associations in survey data.

**Supplementary Information:**

The online version contains supplementary material available at 10.1186/s13690-021-00562-y.

## Background

Reliable and precise estimates of hypertension prevalence are essential to inform health policies at the global, regional, national, and local levels [1–3]. High blood pressure prevalence is one of the harmonized European Core Health Indicators continuously monitored at the European Union (EU) and its member states, using self-reported survey data [1, 2].

There is substantial evidence in epidemiological research on limited validity of self-reports to measure hypertension [4–6]. Survey participants could misclassify their health status and report it as healthy due to inaccurate recall or lack of awareness [4–8]. Several studies have shown that, across EU countries, the awareness of hypertension is still far from perfect (ranging between 33.9 and 82.2%) [5–7, 9, 10] and may be differential among population subgroups [5–7, 9], since some are more prone to reporting errors in surveys.

Incorrect reports of hypertension lead to inaccurate survey inference on prevalence and measures of association, so called misclassification bias [4–6, 8], with implications for public health planning, interventions evaluation, and research. To reduce misclassification bias objective measure of blood pressure through health examination surveys (HES) has thus been recommended [6]. However, objective measurements are more expensive and time-consuming, and represent a higher burden for participants [11]. Surveys with objective measurements are usually implemented in a smaller scale with implications for estimates precision, limited level of disaggregation and subsequent limited subpopulation analysis. Even in high-income countries, frequent HES may not be a feasible substitute of large-scale health interview surveys, and as such, decision makers and researchers still rely highly on self-reports. A possible way to address this issue would be to maintain self-report measures from large-scale surveys with high precision but attempt to adjust it for the potential misclassification bias using more accurate HES data.

Several methods, such as regression calibration, maximum likelihood and Bayesian approaches [12–16] and respective software solutions [14, 15] have been put forward to account for misclassification in this context. However, these are complex and might not be intelligible for the average public health researcher. A more feasible alternative is to consider misclassification as a missing data problem and apply multiple imputation techniques for misclassification error correction (MIME) [12, 17–19]. MIME is deemed simple to use and does not require strong programming skills, since multiple imputation routines are included in all standard statistical software [18]. Another advantage of MIME is that it can be applied to correct in a single step bias in both prevalence estimates and exposure-outcome associations, allowing accounting for differential and non-differential misclassification errors either in outcome or exposure, and using internal and external validation data [12, 17–20].

National Health Interview Survey (INS) [21] represents a key tool for monitoring trends in hypertension and other cardiovascular disease risk factors in Portugal, providing evidence for public health planning and National Health Programs [3, 22]. Since 2014, INS is the Portuguese component of European Health Interview Survey, used for European Core Health Indicators monitoring in the EU. In 2015, Portugal developed its first HES combining self-reported and objectively measured data on hypertension for the same individuals, thus providing an opportunity to investigate the magnitude and direction of misclassification bias in self-reports and the feasibility of statistical adjustments.

Despite extensive methodological research on methods performance in a variety of settings and recently growing attention to misclassification bias in self-reports in epidemiological literature, this issue is still overlooked in public health practice; few studies attempted to assess results robustness in the presence of misclassification or adjust for it [23, 24].

This study aims to illustrate the application of multiple imputation for misclassification bias correction in self-reported hypertension prevalence and prevalence ratios in the INS2014, using data on objectively measured blood pressure from the first Portuguese HES (INSEF).

## Methods

### Study settings

We used data from two population-based surveys: with interview (INS2014) and examination (INSEF) conducted in Portugal in 2014–2015. A detailed description of surveys design, sampling, and data collection has been provided elsewhere [25, 26].

Briefly, INSEF is a cross-sectional study conducted in 2015 by the Instituto Nacional de Saúde Doutor Ricardo Jorge, in partnership with the five Regional Health Administrations, the Regional Secretariats for Health of the Autonomous Regions (Azores and Madeira) and the Norwegian Institute of Public Health. INSEF collected objectively measured and self-reported data on a multi-stage probabilistic sample (*n* = 4911, response rate of 43.9%) of non-institutionalized Portuguese population aged 25–74 years old (yo), through physical examinations and interviews.

INS2014 was developed by Statistics Portugal and Instituto Nacional de Saúde Doutor Ricardo Jorge as an integrated part of European Health Interview Survey, wave 2. It is a cross-sectional study targeting non-institutionalized resident population aged 15 years or over. Survey sample (*n* = 18,204) was selected using multistage stratified design, participation rate was 80.8% [25]. INS2014 collected self-reported data on sociodemographic characteristics, health status and its determinants. For this study, INS2014 sample was restricted to individuals aged 25–74 yo (*n* = 13,937), i.e., the age group available in both surveys.

### Definitions

Self-reported hypertension prevalence was estimated using the following question of INS2014: “*During the past 12 months, have you had any of the following chronic disease or conditions? High blood pressure/Hypertension (Yes/No). Consider disease/conditions even if the symptoms were not present due to medical treatment”*. Individuals who answered “yes” were considered hypertensive.

For INSEF, self-reported hypertension prevalence was defined using two questions: i) “*Do you have any of the following longstanding diseases or conditions: High blood pressure or hypertension? (Yes/No)*” *Consider longstanding disease/conditions which have lasted, or are expected to last, for 6 months or more.* , and if yes, ii) “*Were these conditions diagnosed by a medical doctor? (Yes/No)*”. Individuals who answered positively to both questions were considered hypertensive.

Prevalence of objectively measured high blood pressure was defined as the proportion of those: i) with systolic blood pressure ≥ 140 mmHg, or ii) with diastolic blood pressure ≥ 90 mmHg, or iii) reporting to take prescribed medication to control blood pressure in the 2 weeks prior to the interview. Blood pressure was measured in a sitting position after 5 min of rest using automated measurement device OMROM M6. Three sequential blood pressure measurements were taken on the right arm with one-minute intervals. The average of the 2nd and 3rd readings for systolic and diastolic blood pressure was considered. Medication intake was assessed by the questions: “*During the past 2 weeks, have you used any medicines that were prescribed for you by a doctor?*” and if yes, “*Were the medicines for hypertension?*”

Both surveys collected data on sex, age group, region of residence, urbanization, education, income, health behaviours and healthcare use, using similar questions (Table S1).

### Statistical analysis

Participants’ characteristics and self-reported hypertension prevalence rates were compared between the two surveys using chi-square test. Exploratory analysis of missing data was performed, logistic regression was used to investigate whether probability of objectively measured hypertension being missing is related to observed data.

We used logistic regression imputation method for monotone missing data patterns for misclassification correction. We assumed that objectively measured hypertension was missing at random for INS2014 participants and imputed it using INSEF as auxiliary data. Logistic regression model was fitted on the INSEF sample considering objectively measured hypertension as outcome and self-reported hypertension and set of other covariates as independent variables. Among covariates available in both surveys only statistically significant were included in the final imputation model. Model performance was assessed using area under receiver operating curve (AUC) and Archer-Lemeshow goodness-of-fit test [27]. Fitted model was used to impute “objectively measured” hypertension values in the INS2014 sample. Imputation was based on a set of 100 imputation iterations.

We estimated objectively measured, self-reported and MIME-corrected prevalence of hypertension and respective 95% confidence intervals (95CI) for overall sample and stratified by sex, age group and educational level. Poisson regression models were fitted to estimate prevalence ratios (PR) of self-reported, objectively measured and MIME-corrected hypertension according to sex, age group and educational level. Poisson regression was chosen since it allows to estimate prevalence ratio directly and is recommended as alternative to logistic regression when outcome is not rare [28, 29].

Self-reported and MIME-corrected INS2014 prevalence and prevalence ratios estimates were compared in terms of bias, standard error (SE) and mean squared error (MSE). Bias in self-reported and MIME-corrected estimates were computed using objectively measured INSEF data as gold standard. MSE was estimated as a sum of the variance and the bias squared.

Stata 15.1 was used for data analyses [30]. All analysis presented in the manuscript, including multiple imputation, were performed using sampling weights to account for the complex sample design of INS2014 and INSEF samples. Significance level of 5% was considered.

## Results

### Participants characteristics and surveys comparability

INS2014 and INSEF respondents were similar regarding sociodemographic characteristics (Table S2), which is consistent with the samples being representative of the Portuguese population. Differences between surveys were observed for three of 11 variables. Notably, we observed a higher proportion of INSEF participants reporting to have their blood pressure measured by a health professional in the last 12 months (82.2% vs. 78.1%), and to consume alcohol in the last 12 months (80.1% vs. 73.1%). In contrast, a lower proportion of INSEF participants reported to have consulted a general practitioner in the last 12 months (65.2% vs.74.9%).

### Imputation model

Of potential 11 covariates: self-reported hypertension, sex, age group, region of residence, level of education and practice of physical activity (Table S3) logistic regression model used for MIME included six that were statistically significant. Model showed good fit (Archer-Lemeshow goodness-of-fit test *p*-value = 0.167) and excellent classification accuracy (AUC = 0.92).

### Hypertension prevalence

Self-reported prevalence estimates were similar between both surveys, except for the 55–64 age group, where higher estimates (*p*-value = 0.0288) were obtained for the INSEF sample (Table 1). In both surveys, self-reported prevalence was lower compared to objectively measured; the extent of underestimation varied by population subgroup.

**Table 1:** Prevalence of objectively measured, self-reported and MIME-corrected hypertension stratified by sex, age group and educational level in INSEF and INS2014

	INSEF Self-reported		INS2014 Self-reported		INSEF Objectively measured		INS2014 MIME-corrected	
	p	95CI	p	95CI	p	95CI	p	95CI
**Overall**	25.7	[23.9, 27.5]	24.5	[23.4, 25.7]	36.0	[34.3, 37.7]	35.3	[33.3, 37.3]
**Sex**								
Female	26.1	[24.0, 28.3]	26.8	[25.2, 28.5]	32.7	[30.1, 35.5]	33.2	[30.8, 35.6]
Male	25.1	[22.1, 28.4]	22.0	[20.5, 23.7]	39.6	[36.5, 42.8]	37.6	[34.4, 40.8]
**Age group**								
25–44 years^a^	6.0	[4.6, 7.8]	7.1	[6.0, 8.2]	12.1	[9.9, 14.7]	13.1	[10.6, 15.6]
45–54 years	19.8	[16.7, 23.3]	22.5	[20.2, 25.0]	35.8	[31.3, 40.6]	37.4	[33.1, 41.7]
55–64 years	47.1	[41.4, 53.0]	39.8	[37.0, 42.6]	58.4	[51.4, 65.0]	52.7	[48.1, 57.2]
65–74 years	58.9	[53.0, 64.6]	54.3	[51.2, 57.3]	71.3	[65.7, 76.4]	68.3	[63.9, 72.6]
**Educational level**								
ISCED^b^ 2011 levels 0–1	41.8	[38.2, 45.4]	39.3	[37.3, 41.2]	55.4	[51.9, 58.8]	53.4	[50.0, 56.8]
ISCED 2011 level 2	18.7	[15.4, 22.6]	18.3	[16.0, 20.9]	29.4	[25.0, 34.2]	29.5	[25.1, 33.9]
ISCED 2011 levels 3–4	15.2	[12.2, 18.8]	12.9	[10.9, 15.1]	24.0	[20.6, 27.8]	21.6	[17.4, 25.9]
ISCED 2011 levels 5–8	10.6	[7.8, 14.11]	10.2	[8.4, 12.3]	15.5	[12.6, 18.9]	15.5	[12.3, 18.6]

INSEF – Portuguese Health Examination Survey, INS2014 – National Health Interview Survey 2014, MIME – Misclassification error correction

^a^the age groups 25–34 years and 34–44 years were aggregated in further analyses given a low number of participants reported hypertension

^b^Educational level was grouped according the 2011 International Standard Classification of Education (ISCED) into four categories

Following MIME correction, prevalence estimates increased substantially and approximated to their objectively measured INSEF counterparts. Overall, MIME reduced misclassification bias from 11.5 pp. to 0.7 pp. (Table 2). Bias reduction was observed in all studied population subgroups, yet, for 55–64 yo the difference between INS2014 and INSEF remained considerable after correction (5.7 pp).

**Table 2:** Misclassification bias and mean squared error (MSE) for self-reported and MIME-corrected hypertension prevalence

	INS2014 Bias, self-reported (pp)	INS2014 Bias, MIME-corrected (pp)	INS2014 MSE, self-reported	INS2014 MSE, MIME-corrected
Overall	11.5	0.7	0.0132	0.0002
**Sex**				
Female	5.9	−0.5	0.0036	0.0002
Male	17.6	2.0	0.0309	0.0007
**Age group**				
25–44 years	5.0	−1.0	0.0026	0.0003
45–54 years	13.3	− 1.5	0.0179	0.0007
55–64 years	18.6	5.7	0.0347	0.0038
65–74 years	17.0	3.1	0.0291	0.0014
**Educational level**				
ISCED 2011 levels 0–1	16.1	2.0	0.0261	0.0007
ISCED 2011 level 2	11.1	−0.1	0.0124	0.0006
ISCED 2011 levels 3–4	11.1	2.4	0.0125	0.0011
ISCED 2011 levels 5–8	5.2	0.0	0.0028	0.0003

MSE = variance + bias^2^

Bias is defined as the absolute difference between the INS2014 and INSEF objectively measured prevalence estimates in percentage points (pp)

INSEF – Portuguese Health Examination Survey, INS2014 – National Health Interview Survey 2014, MIME – Misclassification error correction

Regarding prevalence estimates precision (Table S4), when comparing SE for MIME-corrected estimates obtained with larger INS2014 sample and objectively measured ones from the smaller INSEF sample, we observed marginal improvements for 6 population subgroups, while for other 4 corrected estimates were less precise. Gains in terms of MSE after MIME correction were observed in all studied subgroups (Table 2).

### Prevalence ratios

Prevalence ratios of hypertension according to sex, age group and educational level estimated by Poisson regression are summarized in Table 3.

**Table 3:** Prevalence ratios (PR) and corresponding 95% confidence intervals (95CI) of self-reported, objectively measured and MIME-corrected hypertension by sex, age group and educational level in INS2014 and INSEF

	INSEF, self-reported PR[95CI]	INS2014, self-reported PR[95CI]	INSEF, objectively measured PR[95CI]	INS2014, MIME-corrected PR[95CI]
**Sex**				
Female	ref	ref	ref	ref
Male	1.0 [0.8, 1.1]	**0.8** [0.7, 0.9]	**1.2** [1.1, 1.4]	**1.1** [1.0, 1.3]
**Age group**				
25–44 years	ref	ref	ref	ref
45–54 years	**3.3** [2.4, 4.6]	**3.2** [2.7, 3.9]	**3.0** [2.4, 3.7]	**2.9** [2.3, 3.6]
55–64 years	**7.9** [5.7, 10.9]	**5.7** [4.8, 6.7]	**4.8** [3.8, 6.1]	**4.0** [3.3, 5.0]
65–74 years	**9.8** [6.9, 14.0]	**7.7** [6.6, 9.1]	**5.9** [4.9, 7.2]	**5.2** [4.3, 6.4]
**Educational level**				
ISCED 2011 levels 0–1	**4.0** [3.0, 5.3]	**3.8** [3.2, 4.6]	**3.6** [3.0, 4.3]	**3.5** [2.8, 4.3]
ISCED 2011 level 2	**1.8** [1.3, 2.4]	**1.8** [1.4, 2.2]	**1.9** [1.5, 2.5]	**1.9** [1.5, 2.5]
ISCED 2011 levels 3–4	**1.4** [1.0, 2.0]	**1.3** [1.0, 1.6]	**1.6** [1.3, 1.9]	**1.4** [1.1, 1.8]
ISCED 2011 levels 5–8	ref	ref	ref	ref

INSEF – Portuguese Health Examination Survey, INS2014 – National Health Interview Survey 2014, MIME – Misclassification error correction

Comparing men and women based on self-reported INS2014 data, the prevalence ratio was 0.8 [95CI: 0.7, 0.9], indicating lower hypertension prevalence among men. Objectively measured INSEF data pointed out in opposite direction, indicating 1.2-fold increase in prevalence among men (PR = 1.2 [95CI: 1.1, 1.4]), when compared to women. MIME correction produced statistically significant PR estimate of 1.1 [95CI: 1.0, 1.3], closer to the objectively measured.

INS2014 data indicated a 7.7-fold increase in hypertension prevalence [95CI: 6.6, 9.1] in the 65–74 yo group, compared to the reference group (25–44 yo), while a smaller effect of age was estimated with objectively measured INSEF data (PR = 5.9 [95CI: 4.9, 7.2]). Accounting for outcome misclassification through MIME correction produced a PR estimate of 5.2 [95CI: 4.3, 6.4].

INS2014 estimates of prevalence ratios according to educational level were similar to the INSEF, indicating no need for correction. Prevalence ratios from MIME correction remained close to the original ones (Table 3).

After adjustment for confounding, we observed misclassification bias required correction in self-reported prevalence ratio of hypertension by sex but not by age group or educational level. MIME-corrected PR of 1.2 [95CI: 1.0, 1.3] resulting from multivariate Poisson regression (Table S5) was similar to objectively measured in INSEF.

## Discussion

In this study, we successfully applied MIME correction to adjust self-reported hypertension prevalence and prevalence ratios estimates from INS2014, a large-scale population-based study using external data on objectively measured hypertension from the first Portuguese HES as reference.

Our results showed that self-reported data on hypertension, European Core Health Indicator collected by the Portuguese component of the European Health Interview Survey wave 2, is subject to differential misclassification, and, if ignored, it leads to inaccurate inference and misleading scientific conclusions. Notably, for Portuguese aged 25–74 years old, the underestimated prevalence and severity of bias varied among subgroups. Misclassification bias in prevalence estimates were larger among men, older age groups, and less educated people. Such differences might be explained by recall bias, confusion between “controlled” disease and “cure” and differential health-seeking/contact behaviors (e.g. women have more contact with healthcare services during key life events such as pregnancy and thus might be more aware of high blood pressure) [6–8]. While an understanding of the reasons behind these differences is beyond the scope of our work, a more throughout investigation can shed further light regarding which groups are at higher risk of not being correctly diagnosed.

As expected, MIME approach markedly reduced misclassification bias in overall and strata-specific prevalence estimates. INS2014 MIME-corrected estimates were similar to the objectively measured INSEF prevalence for overall sample (35.3% vs. 36.0%) and in all but the 55–64 yo population subgroups. This is likely to be related to the magnitude of bias, with more heavily biased estimates being harder to correct. Furthermore, we identified and accounted for bias in exposure-outcome associations. It has been previously shown that in the presence of differential misclassification of the outcome the measures of association may be biased in any direction (away or towards null) [14]. In our study, compared to objectively measured, INS2014 self-reported data yielded similar prevalence ratio estimates by education level, but overestimated association with age (7.7 vs. 5.9) and considerably underestimated association with sex (0.8 vs. 1.2). In multivariate analysis, only prevalence ratio by sex required correction (0.8 vs. 1.2). MIME approach successfully corrected direction of association with sex in bivariate and multivariate analysis and also corrected magnitude of the associations with age, where bias were less pronounced. It should be noted that smaller bias in multivariate analysis for the self-reported data might be related to distinct directions of bias to the included variables. Overall, these results are in line with previous research, that reported comparable performance of MIME for prevalence estimates and associations correction with both internal and external validation data [19, 20].

INS2014 sample size was approximately 3-fold the INSEF sample, we thus expected MIME-corrected estimates to be more precise than those derived from objectively measured INSEF data. However, we achieved little or no gain in estimates precision. MIME yielded 12–39% smaller SE for prevalence estimates stratified by sex and age group, whereas for prevalence rates by educational level MIME-corrected SE were 3–11% higher compared to INSEF sample. Simulation studies indicate that gains in precision and MSE depend both on the relative sample size of survey and validation datasets and quality of the multiple imputation models [12, 17, 20]. As recommended in literature, we included in the model potential risk factors for hypertension and variables related to misclassification process in the Portuguese context; our model showed excellent discriminating accuracy (AUC = 0.92). However, we were not able to include other, potentially relevant variables (e.g., body mass index), not present in both surveys or not collected with comparable questions and therefore which could not be included in the imputation model. Inclusion of these additional variables could improve MIME performance. In addition, the sample size ratio between two surveys was small, which might also explain the low gain in precision. More pronounced improvements in precision have been reported in the USA, with 17-fold sample size ratio between NHANES and NHIS [20]. Our results were similar to Edwards et al. [17], who used data with 3.4-fold sample size ratio. Although MIME yielded a small gain in precision, we observed considerably lower MSE for corrected estimates than in the original self-reported data due to bias reduction. Even with little or no gain in precision, bias correction in survey estimates may be important for evidence-based decision-making and public health planning. This is particularly relevant when bias is large or changes the direction of the associations, as in our case, where self-reported data completely distorted sex differences in hypertension prevalence, indicating higher amount of disease among women.

Whilst research may be interested solely in prevalence estimates, most frequently the interest is in exposure-outcome associations. MIME has been previously used in different study designs to correct odds ratios, risk ratios, and hazard ratios [15, 17]. In our study, MIME correction was successfully applied to prevalence ratios estimated by Poisson regression. This result corroborates the flexibility of the MIME approach, applicable to several model types. Another advantage of this correction method is that it allows to account for complex sample design [30]. Nowadays, almost all large-scale surveys use complex sample features (stratification, clustering) to reduce data collection cost. Furthermore, multiple imputation might be extended to account simultaneously for misclassification error and missing data [19]. In our study, the proportion of missing data in covariates used for imputation was below 5%, so we excluded item-missing data. Nevertheless, this might be an important aspect to consider in other studies.

Our approach had several limitations. First, we assumed that information on objectively measured hypertension in INS2014 was missing at random (Table S6), and that missingness was not dependent on individuals’ hypertension status. While we cannot formally assess this, a possible selection bias related to hypertension status were minimized by survey design and data collection method, given that INS2014 is representative of Portuguese population [25].

Second, we assumed that misclassification error properties and imputation model are transportable between surveys. Transportability is a critical issue and it arises regardless of the method used to correct estimates for measurement error [12, 20, 31]. When validation data does not correctly reflect the relationship between self-reported and the objectively-measured, more severe bias may be introduced by correction [31]. We extensively investigated surveys comparability for a large number of covariates and demonstrated that proportion of different categories of participants were similar between them. Although the reference period for INSEF and INS2014 survey questions for self-reported hypertension was different *(“past 12 months”* vs. *“Do you have any longstanding…”*) self-reported hypertension prevalence and prevalence ratio estimates were also comparable, which is reassuring.

Third, data collection settings may represent an additional source of bias, affecting the transportability of imputation model. In INSEF, interviews and examinations were conducted by health professionals in primary healthcare facilities, while in INS2014 most participants were interviewed at home (93.1%) and a small proportion participated via self-administrated questionnaire filled in a web application (6.9%) [25]. We were not able to take these differences into account.

Finally, our approach may be applied only if individual-level data on misclassification and good predictors for multiple imputation model are available. If this is not the case and research has no reliable auxiliary data on misclassification error, alternative methods have been proposed [15].

Although external validation data has been successfully used for self-reported estimates correction in different contexts [16, 20, 32], it may be more reliable to use internal validation data for MIME. Therefore, we recommend addressing the issue of self-reported misclassification by conducting a large-scale survey that incorporates collection of objectively measured blood pressure for a random subset of participants. A combined survey will benefit from a large sample size, precision and representativeness of estimates on required levels of disaggregation and, at the same time, provide relevant information on misclassification error associated to self-report, making possible its analysis and correction reducing transportability issues. It also requires less preparation that two separated surveys, thus increasing efficiency. Selection of predictors for the imputation model should be done for each particular case, as misclassification error properties verified in the present study may not hold for forthcoming European Health Interview Survey waves and other surveys in Portugal, other target populations or settings. National and regional HES carried out in several EU member states in the last decades have demonstrated that direction and severity of misclassification bias in self-reported hypertension vary across place [5, 6, 9] and time [7] and thus might seriously compromise comparisons of European Core Health Indicator between EU member states, regardless of efforts to produce comparable health information. Moreover, reliance on hypertension self-report might affect monitoring of time trends and evaluation of health interventions. Portugal and many other EU countries consider cardiovascular disease as priority for action [22]. When health programs targeting high blood pressure and cardiovascular disease risk factors are in place it is reasonable to expect that awareness of risk factors, in particular hypertension, increases over time with programs implementation, leading to changing misreporting patterns. Therefore, collection of objectively measured blood pressure using standardized measurement protocols should be continued to reassess misclassification bias in the European Core Health Indicator and adjust for it in the further research. The method now proposed can be easily extended to other health indicators subject to misclassification for which a gold-standard measures are feasible to be collected by HES, such us obesity, elevated cholesterol levels, smoking, anemia, among others.

## Conclusion

In conclusion, our results support previous research questioning accuracy of self-reported hypertension to estimate hypertension prevalence and exposure-outcome associations in general population and highlight the importance of bias analysis when using self-reported data on hypertension. MIME approach may be useful to assess the robustness of the research conclusions and correct for bias in this instance.

## Supplementary Information

**Additional file 1: Table S1**. Common variables in INS2014 and INSEF used in the study. **Table S2**. Sociodemographic characteristics of INSEF and INS2014 participants aged 25–74 years old. **Table S3**. AUC for imputation logistic regression models. **Table S4**. Standard error (SE) for INS2014 self-reported, MIME-corrected and INSEF objectively measured hypertension prevalence estimates. **Table S5**. Self-reported, objectively measured and MIME-corrected adjusted prevalence ratios of hypertension according to sex, age group and educational level.** Table S6**. Coefficients of logistic regression model for the probability of examination-based hypertension being missing.

## Data Availability

Access to the micro data for the INS2014, Portuguese component of the European Health Interview Survey 2, can be requested from Eurostat via a research contract. INSEF data may be provided upon reasonable request and with permission of Ethical Committee of the Instituto Nacional de Saúde Dr. Ricardo Jorge.

## Abbreviations

AUC: Area under receiver operating characteristic curve
EU: European Union
HES: Health examination survey
GP: General practitioner
95CI: 95% confidence interval
INS: National Health Interview Survey
INSEF: Portuguese Health Examination Survey
ISCED 2011: 2011 International Standard Classification of Education
MIME: Multiple imputation for misclassification error
MSE: Mean squared error
NHANES: The USA National Health and Nutrition Examination Survey
NHIS: The USA National Health Interview Survey
pp: Percentage points
PR: Prevalence ratio
SE: Standard error
