# Supplementary material for: Combining self-reported and objectively measured survey data to improve hypertension prevalence estimates: Portuguese experience
Source: Arch Public Health. 2021 Apr 8;79:45. doi: 10.1186/s13690-021-00562-y (PMC8028082; doi:10.1186/s13690-021-00562-y)
Supplement: Supplementary file 1 — Additional file 1: Table S1. Common variables in INS2014 and INSEF used in the study. Table S2. Sociodemographic characteristics of INSEF and INS2014 participants aged 25–74 years old. Table S3. AUC for imputation logistic regression models. Table S4. Standard error (SE) for INS2014 self-reported, MIME-corrected and INSEF objectively measured hypertension prevalence estimates. Table S5. Self-reported, objectively measured and MIME-corrected adjusted prevalence ratios of hypertension according to sex, age group and educational level. Table S6. Coefficients of logistic regression model for the probability of examination-based hypertension being missing. [file 13690_2021_562_MOESM1_ESM.docx]

Supplementary Material

Table S1. Common variables in INS2014 and INSEF used in the study

| Variable | INS2014 | INSEF |
| --- | --- | --- |
| Sex | Male/Female | Male/Female |
| Age group | Age of participant in complete years at the moment of interview coded into one of 5 age groups 25-34, 35-44, 45-54, 55-64, 65-74 | Age of participant in complete years calculated from birth and interview dates was coded into one of 5 age groups: 25-34, 35-44, 45-54, 55-64, 65-74 |
| Education level | What is the highest level of education that you have completed successfully? (No formal education, Basic 1º cicle ou 2º cicle, Basic 3º cicle, Secondary education, Post-secondary education, Higher education including post-graduate); Other  Recoded according ISCED-11 | What is the highest level of education that you have completed successfully? (No formal education, Basic 1º cicle, Basic 2º cicle, Basic 3º cicle, Secondary education, Post-secondary education, Higher education, Post-graduate (Mcs/PhD), Other  Recoded according ISCED-11 |
| BP measurement | When was the last time you have your blood pressure been measured by health professional? (Within the past 12 months, 1 to less than 3 years, 3 to less than 5 years, More than 5 years, Never) | When was the last time you have your blood pressure been measured by health professional? (Within the past 3 months, 3-5 months ago, 6-11 months ago, 12 or more months ago, Never) |
| GP consultation | When was the last time you consulted GP or family doctor on your behalf?  (Less than 12 months ago/ More than 12 months ago/Never) | When was the last time you consulted GP or family doctor in public primary care? (Consider consultations on your behalf only).  (Less than 12 months ago/ More than 12 months ago/Never) |
| Income | What is the approximate net monthly income (after tax and other deductions) of all persons in your household? Consider all regular monetary souses of income (employment, social benefits, investments etc).  Recoded in quintiles | Would you tell me which group represents your household’s total net monthly income from all these sources after tax and other deductions?  Recoded in quintiles |
| Region of residence | Variable provided in the sampling frame  (North, Centre, Lisbon and Tagus Vale, Alentejo, Algarve, Madeira, Azores) | Variable provided in the sampling frame  (North, Centre, Lisbon and Tagus Vale, Alentejo, Algarve, Madeira, Azores) |
| Urbanization | Variable provided in the sampling frame  (Rural, Urban, Semiurban) | Variable provided in the sampling frame  (Rural, Urban, Semiurban) |
| Smoking | Do you smoke?  (Yes daily, Yes occasionally, No) | Do you smoke?  (Yes daily, Yes occasionally, No) |
| Alcohol consumption | In the last 12 months how often have you had an alcoholic drink of any kind (beer, wine, spirits, liquor, coctails, ets)? (Daily or almost daily, 5-6 days per week, 3-4 days per week, 1-2 days per week, 2-3 days in a month, once a month, less than once a month, Not in last 12 month, I do no drink alcohol, Never in my whole life) | During the past 12 months, did you drink any alcohol (wine, beer, liquor, brandy, etc? (Yes/No) |
| Physical activity | In a typical week, on how many days do you carry out physical activity for at least 10 min continuously? ( indicate 0 if not practicing) | In a typical week, do you engage in any regular physical activity and if yes, for how many days? |

In both surveys level of education was grouped according the 2011 International Standard Classification of Education (ISCED) into four categories: ISCED 0-1 level (No formal education/Basic(1 cycle)/ Basic 2 cycle, ISCED 2 level (Basic 3 cycle), ISCED 3-4 levels (Secondary/Pos-secondary), ISCED 5-8 levels (Higher/Post-graduate) education

Table S2. Sociodemographic characteristics of INSEF and INS2014 participants aged 25-74 years old

|  | INSEF (n=4911) | | INS2014 (n=13937) | | p-value |
| --- | --- | --- | --- | --- | --- |
|  | n | % | n | % |  |
| **Sex** |  |  |  |  | 1.000 |
| Female | 2,646 | 52.5 | 7,733 | 52.5 |  |
| Male | 2,265 | 47.5 | 6,204 | 47.5 |  |
|  |  |  |  |  |  |
| **Age group** |  |  |  |  | 1.000 |
| 25-34 years | 714 | 18.3 | 2,557 | 18.3 |  |
| 25-44 years | 1,849 | 23.5 | 3,188 | 23.5 |  |
| 45-54 years | 1,193 | 22.4 | 3,023 | 22.4 |  |
| 55-64 years | 1,098 | 19.9 | 3,031 | 19.9 |  |
| 65-74 years | 771 | 15.9 | 2,852 | 15.9 |  |
| **Education** |  |  |  |  | 0.2215 |
| ISCED 2011 levels 0-1 | 2,193 | 40.3 | 6,824 | 42.5 |  |
| ISCED 2011 level 2 | 918 | 18.9 | 2,310 | 17.8 |  |
| ISCED 2011 levels 3-4 | 958 | 21.4 | 2,353 | 19.3 |  |
| ISCED 2011 levels 5-8 | 838 | 19.4 | 2,450 | 20.4 |  |
| **Income** |  |  |  |  | 0.5798 |
| 1Q (Low) | 1,092 | 19.8 | 2,784 | 18.0 |  |
| 2Q | 929 | 18.4 | 2,592 | 18.3 |  |
| 3Q | 872 | 20.4 | 2,751 | 20.1 |  |
| 4Q | 837 | 19.8 | 2,833 | 21.1 |  |
| 5Q (High) | 914 | 21.7 | 2,977 | 22.5 |  |
| **Last BP measurement** |  |  |  |  | 0.0003 |
| Less than 12 months | 3,880 | 82.2 | 10,673 | 78.1 |  |
| 12 month or more or never | 895 | 17.8 | 3,225 | 21.9 |  |
| **Last general practitioner (GP) consultation** |  |  |  |  | <0.0001 |
| Less than 12 months | 3,016 | 65.2 | 10,126 | 74.9 |  |
| 12 month or more | 1,812 | 34.8 | 3,805 | 25.1 |  |
| **Urbanization** |  |  |  |  | 0.9419 |
| Rural | 1,397 | 26.4 | 4,990 | 26.2 |  |
| Urban | 3,514 | 73.6 | 8,947 | 73.8 |  |
| **Region of residence** |  |  |  |  | 0.9998 |
| Norte | 777 | 35.4 | 2,105 | 35.4 |  |
| Centro | 706 | 16.2 | 2,374 | 16.3 |  |
| LVT | 650 | 34.8 | 2,354 | 34.8 |  |
| Alentejo | 690 | 4.6 | 1,587 | 4.6 |  |
| Algarve | 644 | 4.2 | 1,963 | 4.2 |  |
| RA Madeira | 695 | 2.5 | 1,870 | 2.5 |  |
| RA Açores | 749 | 2.3 | 1,684 | 2.3 |  |
| **Practice of physical activity at least once a week** |  |  |  |  | 0.9238 |
| Yes | 1,674 | 34.2 | 4,536 | 34.0 |  |
| No | 3,235 | 65.8 | 9,361 | 66.0 |  |
| **Smoking** |  |  |  |  |  |
| Yes | 1,115 | 22.1 | 3,152 | 22.6 |  |
| No | 3,793 | 77.9 | 10,775 | 77.4 |  |
| **Alcohol consumption in last 12 months** |  |  |  |  | <0.0001 |
| Yes | 3,924 | 80.1 | 9,511 | 73.1 |  |
| No | 986 | 19.9 | 4,398 | 26.9 |  |
| p-value for chi-square test to compare participants distribution in INSEF vs. INS2014 | | | | | |

Table S3. AUC for imputation logistic regression models

| Variables in the model | AUC | p-value for goodness of fit test |
| --- | --- | --- |
| Self-reported hypertension | 0.844 | 1.00 |
| Self-reported hypertension, sex, age group | 0.910 | <0.001 |
| Self-reported hypertension, sex, age group, region | 0.913 | 0.6644 |
| Self-reported hypertension, sex, age group, region, urbanization | 0.915 | 0.4258 |
| Self-reported hypertension, sex, age group, region, education | 0.917 | 0.6364 |
| Self-reported hypertension , sex, age group, region, education, income | 0.920 | <0.001 |
| Self-reported hypertension, sex, age group, region, education, BP measurement | 0.917 | 0.1716 |
| Self-reported hypertension, sex, age group, region, education, GP visit | 0.919 | <0.001 |
| Self-reported hypertension sex, age group, region, education, smoking | 0.918 | 0.354 |
| Self-reported hypertension, sex, age group, region, education, alcohol | 0.918 | 0.2024 |
| Self-reported hypertension, sex, age group, region, education, physical activity | 0.920 | 0.167 |
| * GP visit, Blood Pressure measurement, urbanization, income, smoking, alcohol consumption were not statistically significant and were not included in the final model | | |

Table S4. Standard error (SE) for INS2014 self-reported, MIME-corrected and INSEF objectively measured hypertension prevalence estimates

|  | SE  self-reported | SE  MIME-corrected | SE  INSEF obj. measured |
| --- | --- | --- | --- |
|  |  |  |  |
| Overall | 0.0056 | 0.0099 | 0.0099 |
| **Sex** |  |  |  |
| Female | 0.0077 | 0.0134 | 0.0154 |
| Male | 0.0075 | 0.0154 | 0.0179 |
| **Age group** |  |  |  |
| 25-44 years | 0.0056 | 0.0125 | 0.0117 |
| 45-54 years | 0.0114 | 0.0218 | 0.0259 |
| 55-64 years | 0.0134 | 0.0230 | 0.0380 |
| 65-74 years | 0.0144 | 0.0220 | 0.0299 |
| **Education** |  |  |  |
| ISCED 2011 levels 0-1 | 0.0094 | 0.0156 | 0.0177 |
| ISCED 2011 level 2 | 0.0117 | 0.0236 | 0.0230 |
| ISCED 2011 levels 3-4 | 0.0099 | 0.0205 | 0.0184 |
| ISCED 2011 levels 5-8 | 0.0092 | 0.0164 | 0.0157 |

Table S5. Self-reported, objectively measured and MIME-corrected adjusted prevalence ratios of hypertension according to sex, age group and educational level

| Adjusted PR | INS2014, self | | | INSEF, self | | | INSEF, exam-based | | | MIME-corrected | | |
| --- | --- | --- | --- | --- | --- | --- | --- | --- | --- | --- | --- | --- |
| SEX | PR | IC 95 | | PR | IC 95 | | PR | IC 95 | | PR | IC 95 | |
| **Sex** |  |  |  |  |  |  |  |  |  |  |  |  |
| Female | ref |  |  | ref |  |  | ref |  |  | ref |  |  |
| Male | 0.8 | 0.7 | 0.9 | 1.0 | 0.9 | 1.1 | 1.2 | 1.1 | 1.4 | 1.2 | 1.0 | 1.3 |
| **Age group** |  |  |  |  |  |  |  |  |  |  |  |  |
| 35-44 years | ref |  |  | ref |  |  | ref |  |  | ref |  |  |
| 45-54 years | 2.7 | 2.3 | 3.3 | 2.9 | 2.1 | 4.9 | 2.6 | 2.1 | 3.3 | 2.5 | 2.0 | 3.1 |
| 55-64 years | 4.5 | 3.7 | 5.3 | 6.6 | 4.9 | 8.8 | 4.0 | 3.2 | 5.1 | 3.3 | 2.6 | 4.1 |
| 65-74 years | 5.7 | 4.7 | 6.7 | 7.9 | 5.7 | 10.8 | 4.8 | 3.9 | 5.8 | 4.0 | 3.3 | 5.0 |
| **EDUC** |  |  |  |  |  |  |  |  |  |  |  |  |
| ISCED 2011 levels 0-1 | 2.1 | 1.7 | 2.5 | 1.7 | 1.4 | 2.2 | 1.8 | 1.6 | 2.2 | 2.0 | 1.7 | 2.7 |
| ISCED 2011 level 2 | 1.6 | 1.3 | 1.9 | 1.4 | 1.0 | 1.8 | 1.5 | 1.2 | 1.9 | 1.7 | 1.3 | 2.1 |
| ISCED 2011 levels 3-4 | 1.3 | 1.0 | 1.6 | 1.3 | 0.9 | 1.8 | 1.4 | 1.1 | 1.8 | 1.4 | 1.2 | 1.9 |
| ISCED 2011 levels 5-8 | ref |  |  | ref |  |  | ref |  |  | ref |  |  |

Table S6. Coefficients of logistic regression model for the probability of examination-based hypertension being missing

|  | β | CI 95% | | p-value |
| --- | --- | --- | --- | --- |
| Sex |  |  |  |  |
| Male | 0.004709 | -0.07832 | 0.087734 | 0.911 |
| Age group |  |  |  |  |
| 35-44 | -0.02034 | -0.15427 | 0.11358 | 0.766 |
| 45-54 | -0.01355 | -0.20076 | 0.133483 | 0.887 |
| 55-64 | -0.02651 | -0.20736 | 0.15434 | 0.774 |
| 65-74 | -0.03492 | -0.249 | 0.17915 | 0.749 |
| Education |  |  |  |  |
| ISCED 2011 level 2 | -0.15724 | -0.28735 | -0.02713 | 0.018 |
| ISCED 2011 levels 3-4 | -0.20422 | -0.37182 | -0.03663 | 0.017 |
| ISCED 2011 levels 5-8 | -0.05384 | -0.29486 | 0.187177 | 0.661 |
| Region of residence |  |  |  |  |
| Centro | 0.016786 | -0.16972 | 0.203291 | 0.860 |
| LVT | 0.013114 | -0.30659 | 0.332822 | 0.936 |
| Alentejo | 0.026791 | -0.15359 | 0.207172 | 0.771 |
| Algarve | 0.011887 | -0.22249 | 0.246262 | 0.921 |
| RA Madeira | -0.01126 | -0.25872 | 0.236193 | 0.929 |
| RA Açores | 3.33E-05 | -0.43758 | 0.43765 | 1.000 |
| Self-reported hypertension | |  |  |  |
| Yes | -0.09367 | -0.24033 | 0.052987 | 0.210 |
| Urbanization |  |  |  |  |
| Rural | 0.023895 | -0.19796 | 0.245746 | 0.833 |
